# Topical emollient application in term healthy newborns: A systematic review

**DOI:** 10.7189/jogh.12.12002

**Published:** 2022-07-25

**Authors:** Mayank Priyadarshi, Bharathi Balachander, Shuchita Gupta, Mari J Sankar

**Affiliations:** 1 Department of Neonatology, All India Institute of Medical Sciences, Rishikesh, Uttarakhand, India; 2 Department of Neonatology, St. Johns Medical College Hospital, Bangalore, Karnataka, India; 3 World Health Organization; 4 Department of Pediatrics, All India Institute of Medical Sciences, New Delhi, India

## Abstract

**Background:**

This systematic review of randomized trials assessed the effect of emollient application compared to no emollient application in term or near-term healthy newborns.

**Methods:**

We searched MEDLINE via PubMed, Cochrane CENTRAL, Embase, and CINAHL (updated until November 2021), clinical trials databases, and reference lists of retrieved articles. Key outcomes were neonatal mortality, systemic infections, atopic dermatitis, skin condition, and adverse events. Two authors separately evaluated the risk of bias, extracted data, and synthesized effect estimates using relative risks (RR). The GRADE approach was used to assess the certainty of evidence.

**Results:**

We screened 19 243 records and included 16 eligible trials involving 5643 participants. Five trials recruited 3352 healthy newborns (term = 728; gestation ≥35 weeks = 2624); and 11 trials included 2291 term newborns who were ‘at risk’ for developing atopy but were otherwise healthy. We conducted a separate analysis for these two groups of newborns. Emollient application (creams or nut, seed, and vegetable oils) started in the neonatal period and continued for four weeks to two years across studies. Meta-analysis for term healthy newborns suggests that topical emollient application may have little to no effect on atopic dermatitis (RR = 1.29, 95% CI = 0.96-1.72; two trials, 1408 newborns; low certainty evidence). Effects on food allergy (RR = 0.84; 95% CI = 0.42-1.70; one trial, 233 newborns), allergic sensitization to food allergens (RR 1.31; 95% CI 1.03 to 1.68; one trial, 234 newborns) and inhalational allergens (RR = 0.97; 95% CI = 0.44, 2.14; 1 trial, 234 newborns), skin dryness (RR = 0.74, 95% CI = 0.55-1.00; two trials, 294 newborns), and skin problems (RR = 0.92, 95% CI = 0.81-1.05; two trials, 292 newborns) were uncertain. Meta-analysis for ‘at-risk’ newborns suggests that intervention probably lowers the risk of atopic dermatitis (RR = 0.74, 95% CI = 0.63-0.86; 11 studies, 1988 infants; moderate certainty evidence), but may have little or no effect on food allergy and allergic sensitization to food or inhalation allergens. The effect on skin dryness and skin rash was uncertain.

**Conclusions:**

Topical emollient application may not prevent atopic dermatitis in term healthy newborns. There is little data for other skin and allergic outcomes.

**Registration:**

Priyadarshi M, Balachander B, Rao S, Gupta S, Sankar MJ. Use of emollients in term healthy newborns: A systematic review. PROSPERO 2020 CRD42020177437.

A neonate’s skin is a dynamic and complex organ undergoing maturation. It provides UV protection, prevents pathogen invasion, and regulates body temperature [1]. Neonates and infants have thinner skin with a larger body surface area, making them prone to transcutaneous uptake of harmful substances. This may lead to skin injury, sensitization to specific allergens, and loss of epidermal barrier function involved in the mechanism of atopic dermatitis (AD), also called eczema, along with cutaneous inflammation [2]. Food allergy is another manifestation of an allergic disease that occurs on exposure to a specific food, which can be IgE- or non-IgE-mediated.

Emollients are lipid-based products that help soothe, soften, and moisturise the skin. Depending on their application and use, they are classified as bath/wash products or leave-on emollients. The leave-on emollients are available in various forms such as creams, ointments, lotions, oils, gels, sprays, and emulsions for skincare. Emollients are made up of active (ceramide or humectant) and excipient ingredients (emulsifiers). Examples of humectants include glycerol and urea, which help in retaining water in the skin, while ceramides are intracellular lipids found in the stratum corneum [2]. Topical emollients may protect the stratum corneum, increase its hydration, decrease water loss across the skin, and enhance epidermal barrier function. Loss of skin barrier function has been connected to the pathogenesis of AD. Applying emollients before the development of eczema may help in primary prevention of eczema. The plausible harm is that emollients can potentially destroy the acid mantle, which is a key to epidermis function, and their excipients can be absorbed, which may result in contact sensitivity and epidermal injury [3].

Emollients are the mainstay in the treatment of existing eczema [4]. However, their role in the prevention of eczema is not well established. A recent Cochrane review evaluated the effect of skincare interventions (including emollients) for primary prevention of eczema and food allergy in infants (0-12 months) and concluded these interventions to be non-effective for the prevention of eczema [2]. Owing to enhancement of skin barrier function, emollient use has also been evaluated for the prevention of invasive infections and mortality in preterm infants. A systematic review on coconut oil application in preterm infants reported decreased water loss, decreased infection rates, and better growth and skin condition [5]. However, the Cochrane review assessing topical emollient for preventing infection in preterm infants did not find any difference in invasive infection or death in high-income countries, but found some benefit with topical oils in the prevention of invasive infection in low- and middle-income countries [6].

Given the paucity of evidence in term newborn population, we sought to evaluate emollient use in term healthy neonates and to determine the effect of routine use of topical emollient application compared to no emollient application on neonatal and infant outcomes (mortality, invasive infections, and AD) in term healthy newborns.

## METHODS

Randomized controlled trials (RCTs), including cluster or quasi-randomized trials in human neonates, were eligible for this review. The study population was term healthy neonates (babies up to 28 completed days of life). Studies that included only preterm or low birth weight newborns were excluded. Emollients can be used as an additive in bath/wash products or applied on the body as leave-on emollients. Studies were included if one group had received a routine application of leave-on emollients (including oil, cream, ointment, lotion, or moisturizer) and another group did not receive any form of emollient. We included only studies where the intervention was started in the neonatal period. The key outcomes were neonatal mortality (all-cause death in the first 28 days of life); systemic infections (sepsis, pneumonia, or possible serious bacterial infection); AD (meeting the diagnostic criteria of at least one of the established tools, such as UK Working Party diagnostic criteria [7], up to one year of age); skin condition (based on a validated skin assessment score or erythema, rash, itching, oedema, exanthema, dry skin, and urticaria), and adverse events related to emollient application.

### Search methodology

Two authors (MP and BB) independently searched MEDLINE via PubMed, Cochrane Central Register of Controlled Trials (CENTRAL), EMBASE, and CINAHL. The first search was conducted until March 2020 and later updated until November 2021. Searches were limited to human studies. There were no language or publication date restrictions. We also searched related conference proceedings (eg, Pediatric Academic Societies abstracts), clinical trial registries (eg, clinicaltrials.gov), and the reference list of all identified trials/studies. We also contacted researchers and relevant experts in this field for information on unpublished and ongoing trials. The search strategy is provided in Appendix S1 in **Online Supplementary Document****.**

### Data extraction and management

Two authors (MP and BB) extracted data independently using a pilot-tested data collection form to collect information on study setting, design, methods, participants, intervention (eg, type of emollient/oil used, method of application, frequency, duration, and the person implementing the intervention), co-interventions (eg, use of bath oil), outcomes, and treatment effects from each included study.

### Assessment of risk of bias in included studies

Two authors (MP and BB) independently assessed the methodological quality of the selected trials/ studies. For trials, we used the Cochrane Risk of Bias (RoB 2.0) tool [8]. Any disagreements between the authors were resolved by discussion. If required, study authors were contacted for clarification.

### Statistical analysis

Meta-analysis was performed using Stata 15.1 (StataCorp, College Station, Texas, USA). For categorical outcomes, the relative risk (RR) and risk differences (RD) were reported. Adjusted RR was used from the studies where available. For significant findings, the number needed to treat (NNT) was calculated along with 95% confidence intervals. For continuous variables, mean difference (MD) and weighted mean differences (WMD) were reported. We estimated the treatment effects of individual trials and examined heterogeneity between trial results by inspecting the forest plots and quantifying the impact of heterogeneity using the I^2^ statistic. We pooled the results of individual studies using the fixed-effect model if *I^2^* was ≤60%. If we detected significant heterogeneity (*I^2^*>60%), we explored its possible causes. If there was no obvious clinical heterogeneity, we used the random-effects model for meta-analysis. We used the GRADEpro software for assigning the quality of evidence [9].

## RESULTS

**Figure 1:**
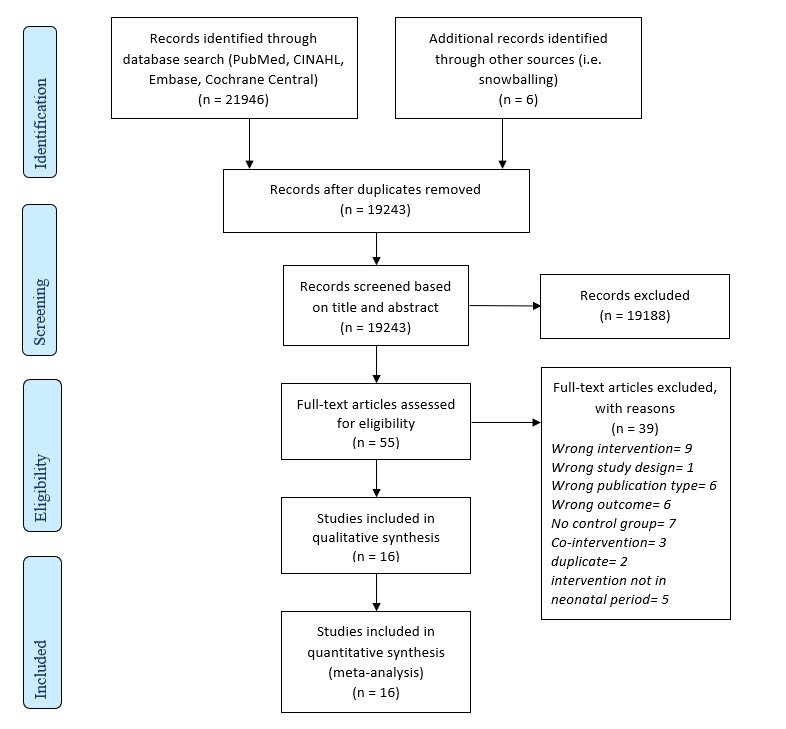
PRISMA flowchart depicting the selection of studies included in the review.

We screened 19 243 records and included 16 studies in this review (**Figure 1****,** **Table 1**). Two studies were published as conference abstracts [15,19]. One trial was available as a study protocol [22], but results could be extracted from a Cochrane review [2]. Five of the 16 trials included 5643 healthy term or near-term newborns, and 11 trials recruited 2291 newborns “at risk” of developing atopy. We analysed the outcomes separately for these two groups of newborns: comparison 1 on emollient application for term, healthy newborns and comparison 2 for emollient application in term, healthy but ‘at risk’ newborns.

**Table 1:** Characteristics of included studies

Study No.	Study author, year	Setting	Study design	Study population/Mean BW/ gestation	Intervention	Control	Outcome parameters of interest	Comments
**Studies included for comparison 1: Emollient application vs no emollient use in term healthy newborns**								
1.	Bartels 2010 [10]	Hospital recruited, community follow up, Germany; HIC	RCT	Healthy full-term newborns with 37 completed weeks of gestation, aged <48 h; Exclusion criteria: sepsis, serious congenital malformations, asphyxia, hydronephrosis, severe intracranial haemorrhage, immunodeficiency, pre-existing skin disease with eruptions covering more than 50% of body surface, relevant skin maceration or inflammation ⁄ irritation, urticaria, acute or chronic diseases with temperatures below 35°C or above 40°C. N = 64 (Group 1 = 16, Group 2 = 16, Group 3 = 16, control = 16) BW = 3.3-3.6 kg/Gest = 40 wk	Four-arm trial: Group 1: WG (washing gel); bathing with pH 5.5 wash gel Group 2: C (cream); bathing with clear water and afterwards topical cream Group 3: WG + C (1&2 both); Bathing with wash gel and topical cream Intervention period: From day 7 till 8 weeks	Group B (bath); Bathing with clear water	Skin condition by neonatal skin condition score (NSCS), trans epidermal water loss, stratum corneum hydration, skin pH, sebum at 8 weeks	Small sample size; high risk of bias; funded by Johnson & Johnson (company products used in the study); data for skin condition not in extractable form; only relevant outcome for this review was diaper dermatitis (data compared between group 2 and control only)
2.	Cooke 2016 [11]	Hospital recruited, community follow up, UK; HIC	RCT	Full term (37 weeks gestation or more), in good health (as determined by the investigator) and were less than 48-72 h old. Exclusion: admitted to Special Care Baby Unit, having phototherapy treatment, had limb defects, non-traumatic impairment of epidermal integrity or evidence of skin disorder at first assessment. N = 115 (olive oil = 38, sunflower oil = 38, no oil = 39) BW = 3.3 kg, Gest = 39 wk	Three-arm trial: Group 1: olive oil Group 2: sunflower oil (Parents applied the oil from the day after the initial assessment. Parents applied 4 drops of oil to their baby’s left forearm, left thigh and abdomen, twice a day) Intervention period: Within 7 d till 4 weeks	No skincare products; water only advocated.	Spectral profile of lipid lamellae, trans-epidermal water loss (TEWL), stratum corneum hydration and pH and recorded clinical observations, at baseline, and 4 weeks post-birth	Pilot study; not powered for important clinical outcomes; data for group 1 and 2 clubbed together as intervention (both considered emollients) and compared with control
3.	Dissanayake 2019 [12]	Hospital recruited, community follow up, Japan; HIC	RCT	Infants born at term. Exclusion criteria: Pre-term birth, Complications due to severe underlying diseases, HBV or HIV positivity of mother at the time of birth, any other inappropriate status as judged by the physician N = 549 (Group 1 = 138, Group 2 = 137, Group 3 = 137, control = 137) Gest = 39 wk, BW = 3.0 kg	Four-arm trial: Group 1: Skin intervention group; emollient 2–3 times/d, after a bath or on clean skin Group 2: Synbiotic intervention group; combination of 0.5 g (7 × 109 CFU/g) of Bifidobacterium bifidum OLB6378 combined with 0.5 g of fructo-oligosaccharides twice a day Group 3: Combined group (1&2 both) Intervention period: birth to 6 mo of age	No intervention (but was not prevented from applying emollients due to ethical reasons)	Atopic dermatitis (Japanese Dermatological Association; and UK Working Party’s Diagnostic Criteria), food allergy, sensitization to food and/or inhalant allergens, Thymus, and activation-regulated chemokine (TARC) score	Data compared between group 1 and control; event rate for AD (as per UK working party definition) used for meta-analysis to maintain uniformity; contamination in the control group
4.	Skjerven 2020 [13]	Hospital recruited, community follow up, Norway and Sweden; HIC	Multi-center RCT	All newborn babies born at a minimum gestational age of 35 weeks Exclusion criteria: pregnancy with more than two fetuses; lack of sufficient Scandinavian language skills; plans to move outside a reasonable travel distance within 1 y postpartum; and severe maternal, fetal, or neonatal disease that could potentially influence adherence to the interventions. N = 2397 (Group 1 = 575, Group 2 = 642, Group 3 = 583, control = 597) Gest = 39 wk, BW = 3.6 kg	Four-arm trial: Group 1: Skin intervention; baths for 5-10 min with added emulsified oil and application of cream to the entire face after the bath on at least 4 d per week, from week 2 to age 8 mo Group 2: food intervention; complementary feeding introduced between 12 and 16 weeks of age in breastfed or formula-fed babies as follows: peanut butter was given for the first time at the scheduled 3-mo clinical follow up investigation, followed by cow’s milk 1 week later, wheat porridge the next week, and finally scrambled eggs in the fourth week of introduction. Group 3: combined (1&2 both) Intervention period: From day 7 until 8 mo	No specific advice on feeding practices or skin care	Primary: Atopic dermatitis assessed at 12 mo of age and food allergy to any intervention allergen assessed at 3 y of age. Secondary: asthma (recurrent bronchial obstruction), food allergy to any other allergen, anaphylaxis, or allergic rhinitis assessed first at 36 mo of age.	Low full protocol adherence of 27% in the skin group and 32% in the food group but 99% adherence in control group; emollient was applied only on face, food allergy is yet to be reported at 3 y
5.	Yonezawa 2017 [14]	Hospital recruited, community follow up, Japan; HIC	RCT	Newborns 1) born at a minimum gestational age of 35 weeks; 2) born to Asian parents; 3) received no medical treatment in the pediatric ward; and 4) newborns who had a mother who was able to speak Japanese Exclusion criteria: not mentioned N = 227 (intervention = 113, control = 114) Gest = 39 wk, BW = 3017 g	Moisturizing skincare as follows: 1) routine bathing every 2 d; and 2) the use of a moisturizer once or more times per day Intervention period: From week 1 till week 12	Common skincare regimen used in Japan as follows: 1) routine bathing daily; and 2) no moisturizer	skin barrier function (transepidermal water loss [TEWL], stratum corneum hydration [SCH], skin pH and sebum secretion) at 3 mo, incidence of skin problems according to parents’ diary reports	Contamination in the control group (use of a moisturizer allowed); unmasked assessment of outcomes; presence of differences in skin barrier function between the two groups at baseline
**Studies included for comparison 2: Emollient application vs no emollient use in at-risk newborns**								
6.	Bellemere 2018 [15]	No information (Europe)	RCT	Newborns at risk aged 2 to 3 weeks with 2 atopic first-degree relatives Exclusion criteria: no information N = 120 (intervention = 60, control = 60) Gest = NA, BW = NA	Use of balm twice a day, cleansing cream and bath oil twice a week from the same brand for 6 mo Intervention period: From 2-3 weeks till 6 mo	No specific intervention	Frequency of AD in the first 6 mo of life (clinical) and at 2 y (phone survey)	Only abstract available; the infants were “at risk” for developing eczema; high risk of bias
7.	Chalmers 2020 [16]	Hospitals, primary care sites and community, UK; HIC	Multi center RCT	Term infants (at least 37 weeks’ gestation) at high risk of developing eczema (ie, at least one first-degree relative with parent reported eczema, allergic rhinitis, or asthma diagnosed by a doctor) were included. Exclusion criteria: preterm birth (birth before 37 weeks’ gestation); a sibling (including twin) randomly assigned in the trial; a severe widespread skin condition that would make detection or assessment of eczema difficult; a serious health issue that would make it difficult for the family to take part in the trial; and a condition that would make the use of emollient inadvisable N = 1394 (intervention = 693, control = 701) Gest = 40 wk, BW = not mentioned	Application of emollient to the child at least once daily to the whole body (excluding the scalp) until the child reached 1 y of age Intervention period: Within 21 d till 1 y	No emollient	Primary: Eczema at age 2 y (defined by UK working party criteria) Secondary: presence of other allergic diseases (ie, parent-reported wheezing and allergic rhinitis; allergic sensitisation to milk, egg, peanut, cat dander, grass pollen, or dust mite at 2 y; parent reported food allergy and parental report of clinical diagnosis of food allergy at 1 and 2 y	74% adherence rate to intervention at 12 mo; the infants were “at risk” for developing eczema; contamination in the control group (15%-18%)
8.	Glatz 2018 [17]	Hospital recruited, community follow up, USA; HIC	RCT	Newborns at risk for developing AD, ie, parent or sibling with a history of AD, asthma, or allergic rhinitis. Exclusion criteria at enrolment included preterm birth (prior to 37 weeks gestation), major congenital anomaly, hydrops fetalis, or significant dermatitis at birth. Exclusion criteria included receiving systemic or topical antibiotics during the study. N = 23 (intervention = 11, control = 12) Gest = not mentioned, BW = not mentioned	Emollient application started within 3 weeks of birth at least once daily to all body surfaces except the scalp and diaper area for 6 mo Intervention period: Within 21 d of life to 6 mo of age	No emollient	Skin barrier parameters, AD development at 6 mo, and bacterial 16S ribosomal RNA gene sequences of cheek, dorsal and volar forearm samples	Pilot study; the infants were “at risk” for developing eczema; not powered enough for important clinical outcomes; age of assessment too early to detect true incidence of AD
9.	Horimukai 2014 [18]	Hospital recruited, community follow up, Japan; HIC	RCT	Infants within the first week after birth at high-risk infants from atopic dermatitis (family history) without treatment with corticosteroids. Exclusion criteria: Infants with skin lesions such as dyskeratosis or bullosis diagnosed by specialists in dermatology; Small-for-gestational-age (<37 weeks); Infants with hepatic disease, convulsion, cardiac disease, haemophilia, diabetes, and auto immune diseases; Inappropriate cases evaluated by doctors N = 118 (intervention = 59, control = 59) Gest = 39 wk, BW = 3.0 kg	The moisturizer was applied at least once daily to the whole-body surface of infants in the intervention group. Intervention period: Within 1 week of life for 32 weeks.	Petroleum jelly (in both intervention and control groups)	Primary: cumulative incidence of AD plus eczema (AD/eczema) at week 32 of life (AD defined based on modification of the United Kingdom Working Party’s criteria) Secondary: allergic sensitization	Modified UK Working Party criteria was used for diagnosis; age of assessment too early to detect true incidence of AD; infants were “at risk” for developing eczema; possible contamination in the control group
10.	Kataoka 2010 [19]	Hospital recruited, unclear setting for follow up, Japan; HIC	RCT	Full-term newborns that have family history of AD within the second degree of kinship Exclusion criteria: not available N = 71 (numbers not mentioned in each group) Population characteristics: not available	The intervention group was instructed not to wash their face with any detergent and to apply prescribed emollient more than once a day. Intervention period: not mentioned	The control group was instructed to do as the parents like.	obvious itchy eczema or mild eczema at 4 mo and 6 mo, transepidermal water loss (TEWL) at the age of 1 week, 1 mo, 4 mo, 6 mo, and food allergy at 6 mo	Full text not available, data extracted from abstract; no details for missing data; infants were “at risk” for developing eczema; AD definition criteria not clear
11.	Lowe 2018 [20]	Hospital recruited, community follow up, Australia; HIC	RCT	Infants with a family history (either parent or older siblings) of allergic disease (asthma, AD, allergic rhinitis/hay fever. or food allergy), less than 4 d of age; and singleton. Exclusion: a) either their parent had known hypersensitivity to any of the ingredients of EpiCeram; b) born premature (<36 weeks); c) required admission into a neonatal special or intensive care nursery; or d) their parents had insufficient English language skills or were not able to comply with all protocol required visits and procedures N = 80 (intervention = 41, control = 39) Gest = 39 wk, BW = 3.3-3.4 kg	Application of approximately 6 g of EpiCeram to the full skin surface of their child twice per day till 6 mo of age. Intervention period: Within 21 d of life till 6 mo.	No emollient	Atopic dermatitis (based on full UK working party criteria), allergic sensitization, Trans epidermal water loss (TEWL), skin pH, hydration and ‘oiliness’ (sebum) at 6 weeks, 6 mo, and 12 mo of age	Contamination in control group (use of other emollients for on average ≥3 d per week during the intervention period occurred in 39% of the control group); infants were “at risk” for developing eczema; outcomes at 12 mo considered for meta-analysis (to look at sustained effects)
12.	McClanahan 2019 [21]	Maternal hospital wards recruited, community follow up, USA / HIC	RCT	Newborn within 21 d considered at high risk for AD development (first-degree relative with history of AD, asthma, or allergic rhinitis) Exclusion criteria: 1) Premature newborn; 2) Major congenital anomaly; 3) Significant dermatitis at birth; (excluding seborrheic dermatitis); 4) Immunodeficiency disorder 5) Serious medical problem making emollient use inadvisable. N = 100 (intervention = 54, control = 46) Gest = NA, BW = NA	Instructed to apply moisturizer (Cetaphil Restoraderm) daily to all body surfaces excluding the scalp and diaper area and to use the cleanser only as needed during bathing. Intervention period: Within 21 d of life till 24 mo.	Control group was given no specific instructions regarding use of emollients except to use emollients of their choice on an as needed basis (emollient use was ~ 45% during first year and 33%-40% during second year)	Cumulative incidence of AD diagnosed at 12 mo by a blinded investigator, adherence with intervention and incidence of treatment-related adverse events	Contamination in control group (use of other emollients ≥5 d per week in 45% of the control group); infants were “at risk” for developing eczema; outcomes at 12 mo considered for meta-analysis (since emollient use was similar, around 40%-50%, during second year in both groups)
13.	NCT03376243[22]	No information about setting; Germany; HIC	Pragmatic RCT	Newborn with a parent or sibling with a history of atopic eczema, allergic rhinitis, or asthma; in overall good health; term-born; mother at least 18 y of age at delivery and capable of giving informed consent	Application of Lipikar Baume AP+ (emollient) and structured parent education; daily application of emollient to the baby's entire body surface area including the face. Intervention period: within 3 weeks of birth until first year	No emollient intervention -only structured parent education	Feasibility, safety and tolerability of the intervention, cumulative incidence of AD at 1 y, trans-epidermal water loss, microbiome diversity, adverse reactions and parent-reported immediate reaction to a known food allergen	Only trial protocol available, results extracted from Cochrane review[2], infants were “at risk” for developing eczema; no information on adherence to intervention.
				Exclusion criteria: 1) Preterm birth; 2) Child previously randomised to this trial; 3) Major congenital anomaly; 4) Significant inflammatory skin disease at birth (except seborrheic dermatitis); 5) Any immunodeficiency disorder or severe genetic skin disorder; 6) Any condition that would make the use of emollients inadvisable or not possible. N = 54 (intervention = 26, control = 28) Gest = NA, BW = 3.5 kg				
14.	Simpson 2014 [23]	Hospital recruited, community follow up, UK; HIC	RCT	Infants at high risk of eczema, defined as having a parent or full sibling who has (or had) physician-diagnosed atopic dermatitis, asthma, or allergic rhinitis Exclusion: born before 37 weeks’ gestation, major congenital anomaly, hydrops fetalis, an immunodeficiency syndrome, a severe genetic skin disorder, or a serious skin condition that would make the use of emollients inadvisable. N = 124 (intervention = 64, control = 60) Gest = not mentioned, BW = not mentioned	Parents in the intervention group were offered a choice of 3 emollients of different viscosities (an oil, a cream/gel, or an ointment). Applied the emollient at least once daily to the baby’s entire body surface, except for the scalp. Intervention period: soon afterbirth (within a maximum of 3 weeks) until 6 mo of age.	No emollient	Cumulative incidence of atopic dermatitis at 6 mo, age of onset of eczema and proportion of transient cases; incidence of emollient-related adverse events, feasibility outcomes	Feasibility study; age of assessment too early to detect true incidence of AD; infants were “at risk” for developing eczema; contamination in the control group (13%)
15.	Techasatian 2021 [24]	Hospital recruited, community follow up, Thailand; UMIC	RCT	Full-term infants (gestational age >37 weeks) were eligible. A high-risk neonate was defined as one having a parent who has (or had) physician-diagnosed AD, asthma, or allergic rhinitis. Exclusion: severe perinatal complications, required neonatal resuscitation or had a serious skin condition at birth. N = 154 (intervention = 77, control = 77) Gest = 38 wk, BW = 3.2 kg	Parents given choice of 5 emollients and instructed to apply the emollient at least once daily to the baby’s entire body surface (excluding the scalp), Intervention period: starting as soon as possible after birth (within a maximum of 3 weeks) and continuing until the infant was 6 mo of age.	Infant skin care advice in booklets on general skin care, and advice to use a mild, fragrance-free cleanser and shampoo, and to avoid using baby wipes.	6-mo cumulative incidence of investigator confirmed AD, age of onset and severity of AD (using the SCORAD), emollient-related adverse events, adherence to intervention	Infants were “at risk” for developing eczema; age of assessment too early to detect true incidence of AD
16.	Thitthiwong 2020 [25]	Hospital recruited, community follow up, Thailand; UMIC	RCT	Healthy, term infants, aged less than 10 weeks old whose parent(s) or sibling(s) had a history of any allergic disease such as atopic dermatitis, asthma, allergic rhinitis, allergic conjunctivitis, food allergy or other allergic conditions Exclusion: Infants known to have major congenital anomalies, immunodeficiency syndrome, any skin disease other than infantile seborrheic dermatitis or neonatal acne, infants whose parents reported regular emollient use before enrolment N = 53 (intervention = 26, control = 27) Gest = NA, BW = 2.2-2.48 kg	Regular application of the hospital formulated emollient named “Cold Cream” all over the body except periorbital and perioral areas at least once daily shortly within 3 to 5 min after bathing and padding dry the baby skin. Intervention period: Within 10 weeks of life (mean 4 weeks) till outcome assessment (9 mo)	No skin care products on the baby skin except using the gentle liquid baby cleansers during bathing and the barrier ointment or cream on diaper areas as needed	Cumulative incidence of AD at 9 mo, mean age of onset of AD, adverse reaction of Cold Cream application and the factors associated with developing AD	Estimated sample size not reached; late initiation of intervention; age of assessment too early to detect true incidence of AD; infants were “at risk” for developing eczema

AD – atopic dermatitis; BW – birth weight; Gest – gestation; HIC – high-income country; NA – not available; UMIC – upper middle-income country, wk – weeks, NA – not applicable

### Study design

All five included studies for comparison 1 were RCTs. One trial employed a two-arm design [14], one employed three arms for two different types of oil [11], and three studies employed four arms for studying the effects of food intervention in a 2x2 factorial design [10,12,13]. For the three-arm study, we combined data for the two emollient groups (olive and sunflower oils), and for the four-arm trials, we used data from “emollient only” and control groups. All 11 trials included for comparison 2 were two-arm parallel-group RCTs.

### Setting

All studies except two were done in high-income countries (Australia, Japan, Germany, Norway, Sweden, UK, and USA). Two studies were conducted in Thailand, an upper-middle-income country [24,25]. No studies were done in low- or lower-middle-income countries. Most studies enrolled neonates in educational hospitals, instructed parents on the method of emollient application followed by the continuation of emollient application in community settings. For evaluation of outcomes, the families were advised for follow-up hospital visits as scheduled in the study. Two studies did not specify the setting [15,19].

### Participants

The review included 16 RCTs involving 5643 newborns. The five trials included for comparison 1 enrolled 3352 term or near-term healthy neonates without any major comorbidities (sickness, serious skin conditions, anomalies, genetic predispositions, etc) [10-14]. The 11 studies included for comparison 2 recruited 2291 healthy term neonates “at risk” for developing eczema. The risk was defined in most studies as having at least one first-degree relative with parent-reported or physician-diagnosed eczema, allergic rhinitis, or asthma.

### Intervention

#### Type of emollient

In the five studies included for comparison 1, one study used topical cream (Baby Caring Facial & Body Cream Penaten, Johnson & Johnson GmbH, Duesseldorf, Germany) after bathing with clear water [10], one study used oil as the preferred emollient, studying the effect of two different types of oil (olive oil and sunflower oil) vs control [11], one study instructed parents to apply Locobase® REPAIR Cream (Daiichi Sankyo, Japan) which contained ceramide, cholesterol, and free fatty acids, after a bath or on clean skin [12]. The fourth study used Ceridal (GlaxoSmithKline Consumer Healthcare, Philadelphia, PA, USA) after bath [13]. One study did not provide details about the emollient used [14].

In the 11 studies included for comparison 2, one study allowed families to choose between Doublebase Gel (Dermal Laboratories, Herts, UK) or Diprobase Cream (Bayer, Berks, UK) [16], one study offered parents a choice of 3 emollients of different viscosities (an oil, cream/gel, or an ointment) with some differences in choices between centres in UK and USA [23], and one study gave parents a choice of five emollients – Ezerra lotion (HOE Pharmaceuticals Sdn. Bhd., Selangor, Malaysia), Eucerin Omega Plus Extra Soothing (Beiersdorf Co., Ltd, Bangkok, Thailand), Eucerin Omega Soothing lotion (Beiersdorf Co., Ltd, Bangkok, Thailand), Physiogel A.I. restoring lipid balm (Stiefel Co., Ltd, Bangkok, Thailand) and LyL hydrating moisturizer (Cosmaprof Co., Ltd, Bangkok, Thailand) [24].

Other studies used Cetaphil Moisturizing Cream (Galderma Laboratories, Fort Worth, TX) [17], an emulsion-type moisturizer (2e [Douhet] emulsion; Shiseido, Tokyo, Japan) [18], ceramide-dominant emollient (EpiCeramTM; PuraCap Pharmaceutical LLC, NJ, U.S.A.) [20], Cetaphil Restoraderm (Galderma, Baie d’Urfé, Montreal, Canada) which contains shea butter as a lipid source, pseudoceramide-5, and 2 FLG breakdown products [21], and Lipikar Baume AP+ [22]. One study formulated an emollient in their hospital containing white petrolatum, stearyl alcohol, propylene glycol, and glycerin, and named it “Cold Cream” [25]. Two studies did not specify the emollient used [15,19]. One study allowed the use of petroleum jelly in the emollient as well as control groups apart from the study intervention product [18].

#### Initiation, dose, and duration of intervention

In all the 16 included studies, the intervention started in the neonatal period (within the first three weeks of life) and continued for varying periods.

In the five studies included for comparison 1, three studies provided intervention for three months or less: one for four weeks [11], one for eight weeks [10], and one for 12 weeks [14]. One study continued the intervention for six months [12] and one for eight months [13].

One study asked parents to apply emollients twice a day [11], one study advised application two to three times a day [12], and one study allowed more than one application of emollient in a day [14]. Two studies instructed parents to apply emollient after bathing but did not specify frequency [10,13]. One study advised emollient application for at least four days per week [13].

In the 11 studies included for comparison 2, five studies advised applying emollients for six months [15,17,20,23,24], one study advised applying for eight months [18] and one study studied nine months of application [25]. Two studies continued the intervention for 12 months [16,22]. The longest duration was present in the study by McClanahan et al. (24 months) [21]. One study did not specify the duration [19]. Nine studies instructed the parents to apply emollient at least once every day, however permitting them to apply more often if they wished. Two studies advised twice daily application of emollients [15,20].

#### Site of emollient application

In the five studies included for comparison 1, one study advised applying emollient on the face after bath [13], the second study emphasized emollient application on cheeks and perioral area but allowed emollient application to other parts of the body on parental discretion [12]. The third study instructed the application of four drops of oil to the baby’s left forearm, left thigh, and abdomen [11]. Two studies did not specify the site of application [10,14]. Parents were asked to apply emollient specifically after bath in two studies [10,13].

In the 11 studies included for comparison 2, emollient was applied to the whole-body excluding scalp in eight studies, while three studies did not specify the site of application [15,19,22].

#### Adherence to intervention

In one of the five studies included for comparison 1, the weekly adherence ranges were 79% to 93% for the olive oil group, 83% to 94% for the sunflower oil group, and 100% for the no oil group [11]. However, 11% to 43% participants in oil groups also used skin products other than the ones prescribed. One study defined reported full protocol adherence as the use of bath oil additive as well as facial cream on at least four days per week being 27%, even though the compliance for an individual product was higher (43% for bath oil additive and 44% for facial cream) [13]. Three studies did not provide data on adherence.

Five of the 11 studies included for comparison 2 reported data on adherence to the intervention, which was defined variably. The adherence was reported to be 88% at three months, 82% at six months, and 74% at 12 months in one study [16] and 72.4% at six months, 66.7% at 12 months, and 40% at 24 months in another [21]. Two studies defined adherence as the application of the cream for ≥5 days per week, which was reported to be 76% [20] and 85% [23] in the intervention arm. One study reported low (one to three days/week; 54%) to moderate (four to six days/week; 46%) adherence to emollient in the intervention group [24].

### Comparison

For comparison 1, all five included studies instructed parents in the comparison group to provide routine skincare as prevalent in their settings. Routine skincare in most of these studies included a bath with clean water but no emollient use. Two studies advised parents not to use emollients in the control group but did not restrict the parents to applying emollients to their infants due to ethical concerns [12,14]. Two studies had almost no contamination in the control group (≤1%) [11,13]. One study did not provide enough information to rule out contamination [10].

For comparison 2, eight of the 11 included studies employed a “no emollient” control, though one study allowed the use of a barrier ointment or cream on diaper area if required [25]. Two studies advised the use of petroleum jelly [18] and emollient of parental choice (as needed) [21], and one study allowed parents to do as they wished (including emollient application) [19].

One study reported no contamination in the comparison group [24] while three studies reported contamination of up to 18% [16], 39% [20] and 13% [23] in their comparison groups. One study mentioned the use of emollient in 45% at six months, 45% at 12 months, and 33% at 24 months in the comparison group [21]. Two studies did not specify the contamination data [18,19]. There was insufficient information in the rest of the studies to clearly rule out contamination in the comparison group.

### Outcomes

None of the included studies reported mortality or systemic infections. The only critical outcome reported in the included studies was AD.

For comparison 1, two studies reported the outcome of atopic dermatitis at 12 months using UK Working Party’s Diagnostic Criteria [7,12,13]. One study reported AD as per Japanese Dermatological Association also, but we only considered the AD incidence reported according to the UK Working Party’s Diagnostic Criteria for meta-analysis to maintain uniformity [12].

For comparison 2, all the 11 included studies reported the outcome of AD at various ages, with the median age of outcome assessment being nine months (ranging from six months to two years): four studies at six months [17,19,23,24], one study at eight months [18], one at nine months [25], one study at 12 months [22] and one at 24 months [16]. Three studies evaluated this outcome at two time points – one study at six and 12 months [20], one at six and 24 months [15], and one at one and two years [21]. For the meta-analysis, we used data at six months in one study (24-month outcome assessed with phone survey and hence, unreliable [15]) and at 12 months in two studies (long-term outcome more meaningful [20] and adherence rate poor after one year [21]). Most of the studies used the UK Working Party diagnostic criteria or a modification of these criteria for diagnosis of atopic dermatitis [7]. One study diagnosed AD using Hanifin and Rajka’s criteria in Thai infants [24,26]. Five studies did not specify the diagnostic criteria for AD [15,17,19,22,23] while one study used atopic dermatitis guidelines by Eichenfield et al. in 2014 [25,27].

Other important outcomes included food allergy and allergic sensitization to food and inhalation allergens.

For comparison 1, one four-arm trial compared the effect of emollient application, synbiotics and both against control (no emollient or synbiotics) and reported food allergy (diagnosed based on questionnaires at 1 year), allergic sensitization to food allergens (based on allergen-specific IgE levels to egg white, ovomucoid, milk) and allergic sensitization to inhalational allergens (based on allergen-specific IgE levels to house dust mite, cat dander) at 9 months of age [12]. We analysed data from emollient and control groups to derive the effect size on the incidence of food allergy,

For comparison 2, one study reported the incidence of food allergy, confirmed either by oral food challenge or an expert allergy panel if oral food challenge was not done at 2 years [16]. One study also provided data on parent-reported immediate reaction (within 2 hours) to a known common food allergen [22]. Three studies reported the outcome of allergic sensitization to food allergens based on allergen-specific IgE levels or masked skin prick tests to milk, egg, and peanut at a median age of two years [16], eight months [18], and 12 months [20]. One study used levels of specific IgE (binding unit of IgE [BUe]/mL) measured with a DLC chip and converted these measurements into CAP-FEIA equivalents (kUA/L) [18]. In absence of a study-defined outcome, we considered the cut-off of >0.35 kUA/L specific-IgE levels to egg white as allergic sensitization based on available literature. Two studies reported the outcome of allergic sensitization to inhalation allergens (cat dander, grass pollen, ryegrass, and dust mite) at one year [20] and two years [16]. The diagnosis was made similarly as described for food allergens.

Other outcomes included skin condition (based on a validated skin assessment score or erythema, rash, dry skin, etc), other skin integrity measurements (trans-epidermal water loss, stratum corneum hydration, skin pH, sebum), and adverse reactions to emollient use (adverse skin reactions, skin infections, and infant slippages during application).

### Risk of bias in included studies

A summary of the risk of bias assessment in the 16 included studies is provided in Appendix S2 in **Online Supplementary Document**. Six studies were at “high” and ten studies had “some concerns” of bias either due to poor adherence to emollient application in the intervention group or contamination (use of emollients) in the control group. Adherence was not reported in nine trials. We judged two studies to be at high risk of bias as these were available in abstract form, restricting the information accessible for most domains [15,19].

### Effects of interventions

The results are summarized separately for the healthy term or near-term newborns and for ‘at-risk’ newborns (**Table 2 and 3**).

**Table 2:** Summary of findings: Topical emollient application compared to no emollient application in term, healthy newborns

			Anticipated absolute effects (95% CI)		
**Outcome**	**Nº of participants (studies)**	**Relative effect (95% CI)**	**Risk with no emollients**	**Risk difference with emollients**	**Certainty**
Atopic dermatitis	1408 (2 RCTs)	RR = 1.29 (0.96-1.72)	98 per 1000	28 more per 1000 (4 fewer to 71 more)	Low*
Food allergy	233 (1 RCT)	RR = 0.84 (0.42-1.70)	130 per 1000	21 fewer per 1000 (76 fewer to 91 more)	Very low†
Allergic sensitization (food allergens)	234 (1 RCT)	RR = 1.31 (1.03-1.68)	461 per 1000	143 more per 1000 (14 more to 313 more)	Very low‡
Allergic sensitization (inhalation allergen)	234 (1 RCT)	RR = 0.97 (0.71-1.33)	96 per 1000	3 fewer per 1000 (28 fewer to 32 more)	Very low†
Skin dryness	294 (2 RCTs)	RR = 0.74 (0.55-1.00)	440 per 1000	114 fewer per 1000 (198 fewer to 0 fewer)	Very low§
Skin problems (redness, erythema, rash, skin breakdown or as assessed with Neonatal Skin Condition Score)	292 (2 RCTs)	RR = 0.92 (0.81-1.05)	679 per 1000	54 fewer per 1000 (129 fewer to 34 more)	Very low‖

CI – confidence interval, RCT – randomized controlled trial, RR – relative risk

*Downgraded by two levels due to serious risk of bias (most of the pooled effect provided by studies at moderate risk of bias) and serious imprecision (wide confidence interval crossing the line of no effect).

†Downgraded by three levels due to serious risk of bias (most of the pooled effect provided by studies at moderate risk of bias) and very serious imprecision (wide confidence interval crossing the line of no effect and less than 30 events and less than 300 participants).

‡Downgraded by three levels due to serious risk of bias (most of the pooled effect provided by studies at moderate risk of bias) and very serious imprecision (wide confidence interval crossing the line of no effect and less than 300 participants).

§Downgraded by three levels due to very serious risk of bias (most of the pooled effect provided by studies at high risk of bias) and very serious imprecision (wide confidence interval crossing the line of no effect and less than 300 participants).

‖Downgraded by three levels due to very serious risk of bias (most of the pooled effect provided by studies at high risk of bias) and serious imprecision (less than 300 participants).

**Table 3:** Summary of findings: Topical emollient application compared to no emollient application in ‘at-risk’ newborns

			Anticipated absolute effects (95% CI)			
**Outcome**	**Number of participants (studies)**	**Relative effect (95% CI)**	**Risk with no emollients**		**Risk difference with emollients**	**Certainty**
Atopic dermatitis	1988 (11 RCTs)	RR = 0.74 (0.63-0.86)	284 per 1000	74 fewer per 1000 (105 fewer to 40 fewer)		Moderate*
Food allergy	1147 (3 RCTs)	RR = 1.12 (0.84-1.48)	123 per 1000	15 more per 1000 (20 fewer to 59 more)		Low†
Allergic sensitization (food allergens)	1061 (2 RCTs)	RR = 0.97 (0.69-1.36)	92 per 1000	3 fewer per 1000 (28 fewer to 33 more)		Low†
Allergic sensitization (inhalation allergen)	1115 (1 RCT)	RR = 1.47 (0.93-2.33)	51 per 1000	24 more per 1000 (4 fewer to 68 more)		Low†
Skin dryness	52 (1 RCT)	RR = 0.41 (0.12-1.36)	296 per 1000	175 fewer per 1000 (261 fewer to 107 more)		Very low‡
Skin rash	118 (1 RCT)	RR = 0.86 (0.31-2.40)	119 per 1000	17 fewer per 1000 (82 fewer to 166 more)		Very low‡

CI – confidence interval, RCT – randomized controlled trial, RR – relative risk

*Downgraded by one level due to serious risk of bias (most of the pooled effect provided by studies at moderate risk of bias).

†Downgraded by two levels due to serious risk of bias (most of the pooled effect provided by studies at moderate risk of bias) and serious imprecision (wide confidence interval crossing the line of no effect).

‡Downgraded by three levels due to very serious risk of bias (most of the pooled effect provided by studies at high risk of bias) and serious imprecision (wide confidence interval crossing the line of no effect and less than 30 events and less than 300 participants).

#### Comparison 1. Emollient application vs no emollient application for term healthy newborns

**Figure 2:**
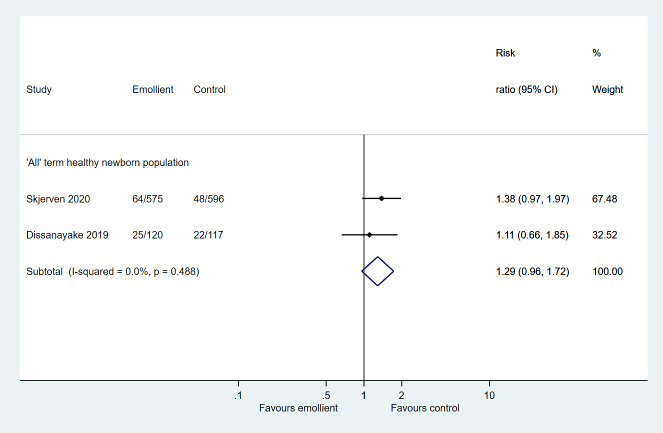
Forest plot for Comparison 1: Emollient application vs no emollient use in term, healthy newborns. Outcome: Incidence of atopic dermatitis (AD).

For AD as the primary outcome, there was little or no difference in the incidence of AD at 12 months of age (RR = 1.29; 95% CI = 0.96-1.72; 2 studies, 1408 participants; low certainty evidence) (**Figure 2**).

For the secondary outcomes there was little data to determine the effect of the topical emollient application on food allergy (RR = 0.84; 95% CI = 0.42-1.70; 1 study, 233 infants; very low certainty evidence), allergic sensitization to food allergens (RR = 1.31; 95% CI = 1.03-1.68; 1 study, 234 participants; very low certainty evidence) or allergic sensitization to inhalational allergens outcome (RR = 0.97; 95% CI = 0.44-2.14; 1 study, 234 participants; very low certainty evidence).

**Figure 3:**
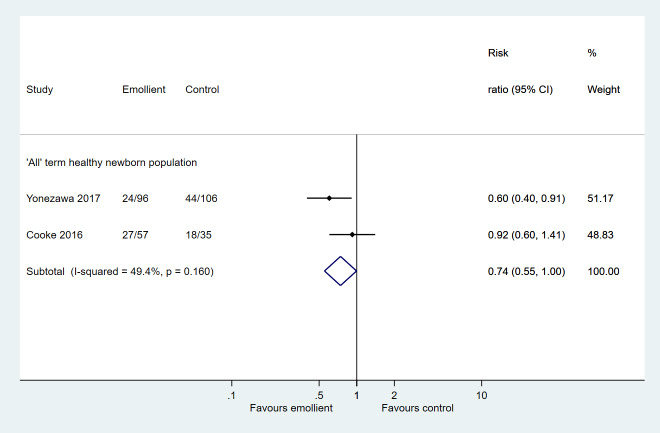
Forest plot for Comparison 1: Emollient application vs no emollient use in term, healthy newborns. Outcome: skin dryness.

Two studies reported skin dryness assessed clinically at four weeks of age [11] and using stratum corneum hydration (<33 percentiles) at 3 months of age [14]. Despite pooling data from both studies, there was little data to determine the effect of the intervention on skin dryness (RR = 0.74, 95% CI = 0.55-1.00; 2 studies, 294 participants; very low certainty evidence) (**Figure 3**).

**Figure 4:**
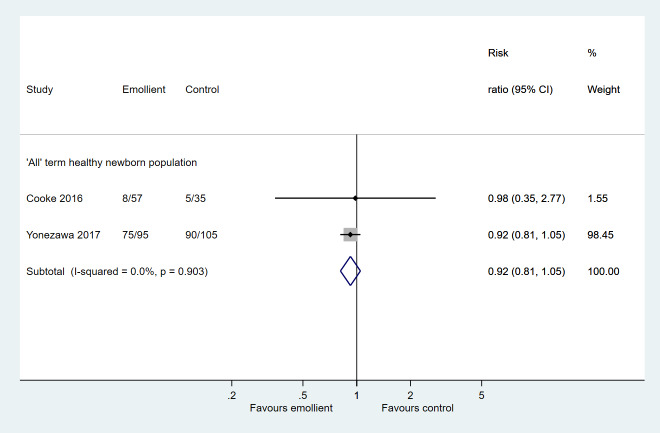
Forest plot for Comparison 1: Emollient application vs no emollient use in term, healthy newborns. Outcome: Skin problems.

Two studies reported skin problems; one reported on parent-recorded redness, erythema, and breakdown in infants’ skin diaries between 5-12 weeks [14] and another on presence of any rash at 4 weeks assessed by midwife using Neonatal Skin Condition Score (NSCS) [11]. Despite pooling, there was little data to conclude an effect of the intervention on skin problems (RR = 0.92, 95% CI = 0.81-1.05; 2 studies, 292 infants; very low certainty evidence) (**Figure 4**).

Three of the five included studies for comparison 1 reported data on adverse events. One four-arm trial (food, skin, combined, and control) recorded any adverse events (including skin reactions) weekly in electronic diaries over the 26 weeks intervention period [13]. The incidence of reported skin reactions (itching, oedema, exanthema, dry skin, and urticaria) was similar in the intervention and comparison groups. The authors reported that nine participants stopped using facial cream while eight stopped bath oil use due to infantile folliculitis or acne (n = 2), seborrhoea (n = 3), worsening of atopic dermatitis (n = 6), and unspecific skin reactions (n = 6). This study also reported a similar incidence of impetigo (n = 9) and hospital admissions (skin = 6/575, food = 9/642, combined = 11/583, and control = 10/596) across all four randomization groups. In the same trial, one incidence of infant slippage (no injury) was reported among 575 infants in the skin intervention group while none was reported in other groups (food, combined, and control).

One trial (Wash Gel, Cream, WG+C, and Control) reported the NSCS at baseline and post-intervention (week 8) in all the four arms and found no significant difference across the groups [10]. The third study did not report any adverse events related to the emollient application [12].

#### Comparison 2. Emollient application vs no emollient application for ‘at-risk’ newborns

A total of 11 studies were included for this comparison (**Table 1**).

**Figure 5:**
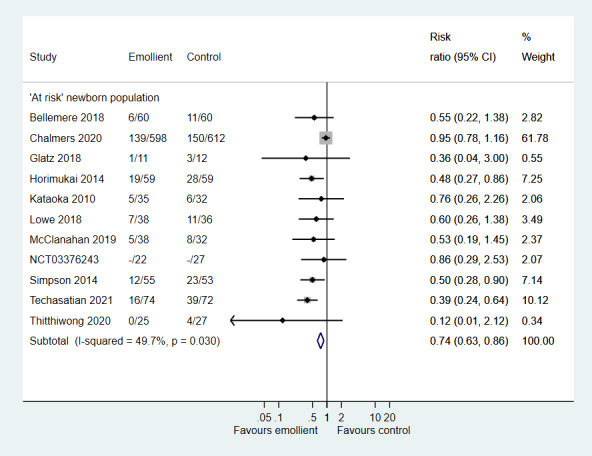
Forest plot for Comparison 2: Emollient application vs no emollient use in at-risk newborns. Outcome: Incidence of Atopic Dermatitis

The incidence of atopic dermatitis was lower in the intervention group (RR = 0.74, 95% CI = 0.63-0.86; 11 studies, 1988 infants; moderate certainty evidence) (**Figure 5**).

For food allergy as the secondary outcome, there was little data to ascertain an effect at 2 years of age (RR = 1.47; 95% CI = 0.93-2.33; 1 study, 1115 participants; low certainty evidence). There were no cases of parent-reported immediate reaction (within two hours) to a known common food allergen (one study, 41 participants; low certainty evidence).

**Figure 6:**
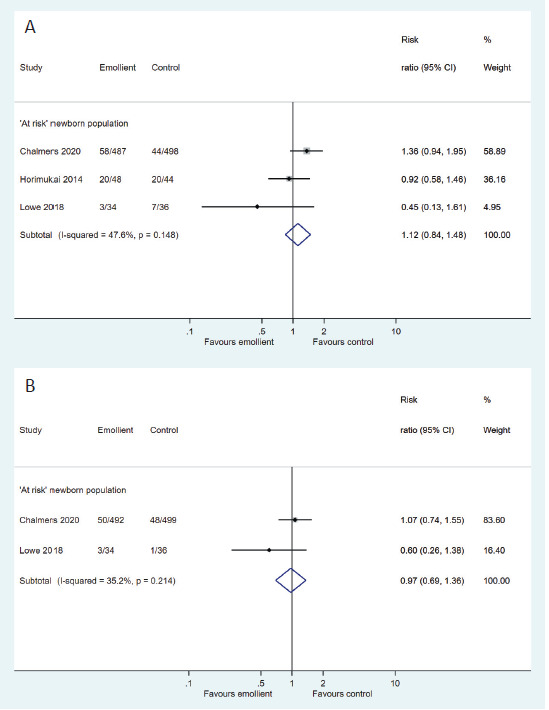
Forest plot for Comparison 2: Emollient application vs no emollient use in at-risk newborns. **Panel A.** Outcome: Allergic sensitization to food allergens. **Panel B.** Outcome: Allergic sensitization to inhalation allergens.

There was paucity of data on the effect of allergic sensitization to food (RR = 1.12, 95% CI = 0.84-1.48, 3 studies, 1147 participants; low certainty evidence) and inhalation allergens (RR = 0.97; 95% CI = 0.69-1.36; two studies, 1062 participants; low certainty evidence) (**Figure 6**, Panels A and B).

There was very little data on skin dryness at nine months of age (RR = 0.41; 95% CI = 0.12-1.36, one study, 52 participants; very low certainty evidence) and other skin conditions like skin rash (without pruritus) at 8 months (RR = 0.86; 95% CI = 0.31-2.40; one study, 118 participants; very low certainty evidence). Both outcomes were assessed clinically by a dermatologist.

For adverse events, one study reported doctor-diagnosed or parent-reported skin infections which included impetigo and unspecified bacterial, viral, or fungal skin infections during the first year [16]. There was a trend towards higher skin infections in the intervention group (RR = 1.34, 95% CI = 1.00-1.80, one study, 1174 participants; not graded). The mean number of skin infections per child was also higher in the emollient group (mean±SD = 0.23 ± 0.68 vs 0.15 ± 0.46; adjusted incidence rate ratio = 1.55, 95% CI = 1.15-2.09). The same study documented similar, though rare occurrences parent-reported infant slippages within an hour of applying emollients (3% vs 2%, 1168 participants; adjusted RR = 1.37, 95% CI = 0.63-2.97). There was no serious injury or admission to the hospital due to any of the reported slippages. Another study reported a significantly increased proportion of infants who had skin reactions (miliaria rubra/pustulosa, benign cephalic pustulosis, and impetigo) in the intervention group (31/74), compared to the control group (2/38) [24].

One study reported a similar incidence of milia, miliaria, acne, erythema toxicum, skin infections, diaper dermatitis, and hypersensitivity reactions in intervention and control groups [21]. One study did not report any emollient-related adverse skin reactions in the intervention group, but emollient use was temporarily stopped in three infants due to suspected contact dermatitis which was later continued when this was judged to be unrelated to the intervention [18].

The remaining three studies did not report any adverse events related to emollient use [20,23,25].

## DISCUSSION

We evaluated the effect of topical leave-on emollient use starting in the neonatal period on atopic dermatitis and other allergic manifestations in infancy. For term healthy newborns, evidence suggests topical emollients may have little to no effect on AD while the effect on food allergy, allergic sensitization to food or inhalation allergens, skin dryness, or skin problems was uncertain. No adverse effects were reported with emollient use. For term newborns “at risk” of AD, evidence suggests that interventions probably lower the risk of AD but may have little or no effect on food allergy and allergic sensitization to food or inhalation allergens. The effect on skin dryness and other skin problems was uncertain.

The disparity in emollient effect between healthy and “at-risk” newborns found in our review is also highlighted by two recent reviews [2,28]. While the Cochrane review did not find emollients during first year of life to be effective for AD prevention [2], Zhong et al. [28] concluded emollients during infancy might prevent AD, particularly in if used continuously with “at-risk” infants. This difference in review results may be related to the nature of question (any skincare intervention vs emollient application), population characteristics (age group 0-12 months vs 0-6 weeks), meta-analysis method (individual patient data vs aggregative data), and AD definition (more inclusive in The Cochrane review). In the review by Zhong et al., the beneficial effect of emollients was limited to the subgroup of “at-risk” infants, similar to our review. Infants who later develop AD have been shown to have higher trans-epidermal water loss (TEWL) during early infancy [29]. Emollient application, started in first few weeks of life, may help protect skin barrier function in such infants. The compliance to emollient use is expected to be better in families with a history of AD, which could have also added to the preventive effect observed in “at-risk” infants. We included all studies on emollient application that were also included in these two reviews, though we did not include AD outcome data from one study owing to its unreliability (parent-reported AD in questionnaire at two years after initial three-month emollient use) [14].

The variations in emollient effect observed across studies can be explained by several factors: included population, criteria and timing of AD diagnosis, type of emollients used, contamination of control groups, etc. The infants studied in the review were from diverse geographical locations with different genetic predispositions. We presented the results separately for healthy and “at-risk” newborns, but the trials enrolling healthy newborns did not assess the predisposition to AD and some healthy newborns might have been “at-risk” for AD. There was heterogeneity in the diagnostic criteria and timing of AD assessment across the studies, which might have affected the results. For example, one study showed a significantly different incidence of AD in the study population as well as across groups when using Japanese Dermatological Association’s diagnostic criteria compared to the UK Working Group’s [12]. There was also a wide variation in the type and composition of emollients in the included trials and one study included a co-intervention of oil bath, which may have different effects compared to emollient use alone. While most emollient products are lipid-based and provide skin barrier protection, there might be different impacts on newborn skin with different compositions of products [11,17]. For example, sunflower oil, compared to mustard oil, induces faster decline in skin pH and may result in reduced mortality among newborns [30,31]. Considering the type and composition of emollient is important designing future trials.

This review tried to determine the effectiveness of topical leave-on emollient application in improving skin outcomes in term healthy neonates. A rigorous methodology was followed, with an all-inclusive literature search and no language filters. Though there were 16 included trials in this review, only five recruited healthy term or near-term newborns, none of which reported mortality or invasive infections, only two reported the primary outcome of atopic dermatitis and single or very small studies reported other important outcomes. Detailed information was not available for three studies (either available as conference abstracts or study protocol). Most studies were at serious or very serious risk of bias. Hence, there is a need for further well-designed trials on emollient application in term, healthy newborns.

## CONCLUSIONS

Topical emollient application may not be protective for AD in term healthy newborns. There is limited data on other outcomes to support the routine use of topical emollients in these newborns. Large, well-designed trials are required to assess the effectiveness of emollient application in term healthy newborns

## Additional material


Online Supplementary Document
