# Supplementary material for: Topical emollient application in term healthy newborns: A systematic review
Source: J Glob Health. 2022 Jul 25;12:12002. doi: 10.7189/jogh.12.12002 (PMC9308984; doi:10.7189/jogh.12.12002)
Supplement: Online Supplementary Document [file jogh-12-12002-s001.pdf]

ONLINE SUPPLEMENTARY DOCUMENT

Topical Emollient Application for Term Healthy Newborns: A Systematic Review

Appendix S1. Search strategy

We used the following search terms for MEDLINE: (Newborn OR infant OR neonat*) AND (emollient OR oil OR cream OR lotion OR ointment OR dermatological agent). Similar terms were used for searching the following databases: Cochrane Central Register of Controlled Trials (CENTRAL), EMBASE, and CINAHL.

Appendix S2. Risk of bias in included studies

A summary of the risk of bias assessment in the 16 included studies is depicted in Figure S1 and Figure S2. Nine out of 16 trials were judged to be at high risk of bias, with most of the bias arising in the domain of deviation from intended interventions. All studies were either at ‘high risk’ (six trials) or ‘some concern’ (10 trials) of bias for this domain, either due to poor adherence to emollient application in the intervention group or contamination in the control group (use of emollients). Adherence was not reported in nine trials. Bellemere 2018 and Kataoka 2010 were available in abstract form, restricting the information accessible for most domains, and were judged to be at high risk of bias.

Figure S1. Risk of bias “traffic light” plots: review authors' judgments about each risk of bias item for each included study

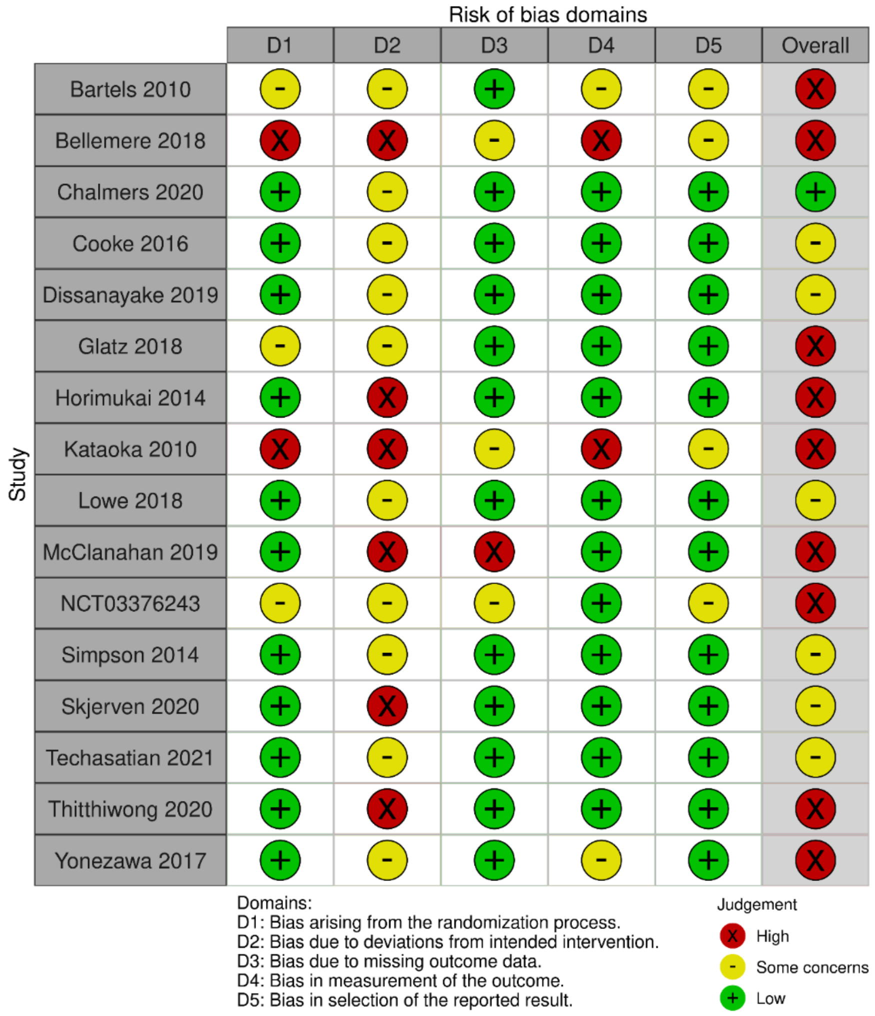

Figure S2. Risk of bias “weighted bar plots”: review authors' judgments about each risk of bias item presented as percentages across all included studies

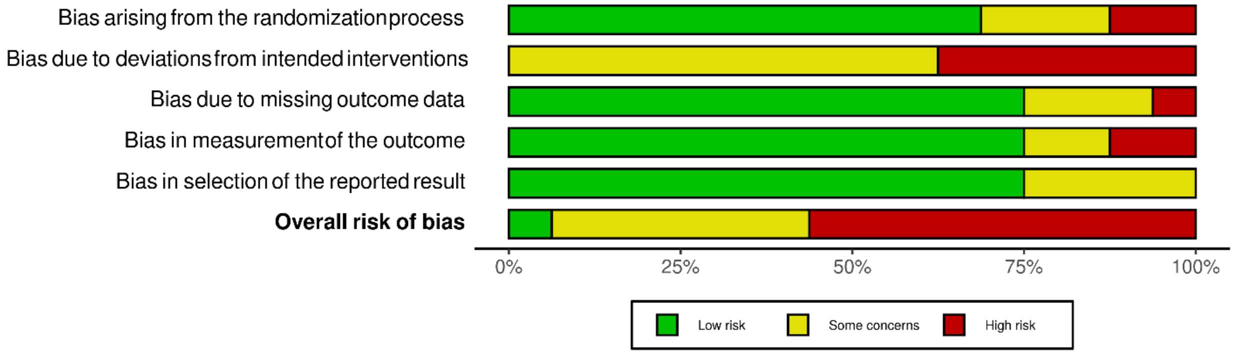

Table S1a. GRADE table: Topical emollient application vs. no emollient application in term, healthy newborns

Certainty assessment							№ of patients		Effect		Certainty	Importance
№ of studies	Study design	Risk of bias	Inconsistency	Indirectness	Imprecision	Other considerations	Topical emollients application	No emollient application	Relative (95% CI)	Absolute (95% CI)		
**Atopic dermatitis**												
2	randomised trials	serious [a]	not serious	not serious	serious [b]	none	89/695 (12.8%)	70/713 (9.8%)	**RR 1.29** (0.96 to 1.72)	**28 more per 1000** (from 4 fewer to 71 more)	⨁⨁◯◯ LOW	CRITICAL
**Food allergy**												
1	randomised trials	serious [a]	not serious	not serious	very serious [b,c]	none	13/118 (11.0%)	15/115 (13.0%)	**RR 0.84** (0.42 to 1.70)	**21 fewer per 1000** (from 76 fewer to 91 more)	⨁◯◯◯ VERY LOW	IMPORTANT
**Allergic sensitization – Food allergens**												
1	randomised trials	serious [a]	not serious	not serious	very serious [b,d]	none	72/119 (60.5%)	53/115 (46.1%)	**RR 1.31** (1.03 to 1.68)	**143 more per 1000** (from 14 more to 313 more)	⨁◯◯◯ VERY LOW	IMPORTANT
**Allergic sensitization. Inhalation**												
1	randomised trials	serious [a]	not serious	not serious	very serious [b,c]	none	11/119 (9.2%)	11/115 (9.6%)	**RR 0.97** (0.44 to 2.14)	**3 fewer per 1000** (from 54 fewer to 109 more)	⨁◯◯◯ VERY LOW	IMPORTANT
**Skin condition. Dryness**												
2	randomised trials	very serious [e]	not serious	not serious	very serious [b,d]	none	51/153 (33.3%)	62/141 (44.0%)	**RR 0.74** (0.55 to 1.00)	**114 fewer per 1000** (from 198 fewer to 0 fewer)	⨁◯◯◯ VERY LOW	IMPORTANT
**Skin condition. Skin problems**												
2	randomised trials	very serious [e]	not serious	not serious	serious [d]	none	83/152 (54.6%)	95/140 (67.9%)	**RR 0.92** (0.81 to 1.05)	**54 fewer per 1000** (from 129 fewer to 34 more)	⨁◯◯◯ VERY LOW	IMPORTANT

CI: Confidence interval; **RR:** Risk ratio

Explanations

a. Most of the pooled effect provided by studies at moderate risk of bias
b. Wide confidence interval crossing the line of no effect.
c. Less than 30 events and less than 300 participants.
d. Less than 300 participants.
e. Most of the pooled effect provided by trials at high risk of bias

Table S1b: Topical emollient application vs. no emollient application in 'at-risk' newborns

Certainty assessment							№ of patients		Effect		Certainty	Importance
№ of studies	Study design	Risk of bias	Inconsistency	Indirectness	Imprecision	Other considerations	the routine use of emollients	no emollients	Relative (95% CI)	Absolute (95% CI)		
**Atopic dermatitis -At risk newborns**												
11	randomised trials	serious	not serious	not serious	not serious	none	210/993 (21.1%)	283/995 (28.4%)	**RR 0.74** (0.63 to 0.86)	**74 fewer per 1000** (from 105 fewer to 40 fewer)	⊕⊕⊕◯ MODERATE	CRITICAL
**Food allergy -At risk newborns**												
1	randomised trials	serious	not serious	not serious	serious [a]	none	41/547 (7.5%)	29/568 (5.1%)	**RR 1.47** (0.93 to 2.33)	**24 more per 1000** (from 4 fewer to 68 more)	⊕⊕◯◯ LOW	CRITICAL
**Allergic sensitization to food allergen -At-risk newborns**												
3	randomised trials	serious	not serious	not serious	serious [a]	none	81/569 (14.2%)	71/578 (12.3%)	**RR 1.12** (0.84 to 1.48)	**15 more per 1000** (from 20 fewer to 59 more)	⊕⊕◯◯ LOW	CRITICAL
**Allergic sensitization to Inhalation allergen- At risk newborns**												
2	randomised trials	serious	not serious	not serious	serious [a]	none	53/526 (10.1%)	49/535 (9.2%)	**RR 0.97** (0.69 to 1.36)	**3 fewer per 1000** (from 28 fewer to 33 more)	⊕⊕◯◯ LOW	CRITICAL
**Skin condition. Dryness-At risk newborns**												
1	randomised trials	very serious [e]	not serious	not serious	very serious [a,c]	none	3/25 (12.0%)	8/27 (29.6%)	**RR 0.41** (0.12 to 1.36)	**175 fewer per 1000** (from 261 fewer to 107 more)	⊕◯◯◯ VERY LOW	CRITICAL
**Skin condition. Skin problems-At risk newborns**												
1	randomised trials	serious [b]	not serious	not serious	very serious [a,c]	none	6/59 (10.2%)	7/59 (11.9%)	**RR 0.86** (0.31 to 2.40)	**17 fewer per 1000** (from 82 fewer to 166 more)	⊕◯◯◯ VERY LOW	CRITICAL

CI: Confidence interval; **RR:** Risk ratio

Explanations

a. Wide confidence interval crossing the line of no effect.
b. Most of the pooled effect provided by studies at moderate risk of bias
c. Less than 30 events and less than 300 participants.
d. Less than 300 participants.
e. Most of the pooled effect provided by trials at high risk of bias
